# Pleural Effusion Presenting in a Young Man With Behcet’s Disease

**DOI:** 10.7759/cureus.10273

**Published:** 2020-09-06

**Authors:** Hashaal F Alkhurassi, Mohammed R Ocheltree, Ahlam Alsomali, Reem A Alqunfoidi, Asmaa Saadallah

**Affiliations:** 1 Internal Medicine, International Medical Center, Jeddah, SAU; 2 Internal Medicine/Geriatric, International Medical Center, Jeddah, SAU; 3 Internal Medicine/Hematology, International Medical Center, Jeddah, SAU

**Keywords:** rare cause of pleural effusion, behcet’s syndrome, hepatic vein thrombosis

## Abstract

Behcet's disease (BD) is a rare multisystem chronic vasculitis of variable clinical presentation and unknown origin. Pulmonary involvement in BD is uncommon, with pleural effusion being an even rarer and difficult to diagnose manifestation. Herein, we report a challenging case of a young man who presented with recurrent pleural effusion and hepatic vein thrombosis and a recent history of papilledema with idiopathic intracranial hypertension. The patient was hospitalized for diagnostic and therapeutic thoracocentesis. Biochemistry and cytological analysis showed an exudative aspect of the collected pleural fluid with lymphocyte-dominated cytology. A multidisciplinary discussion was held, and thorough investigation was carried out to rule out malignant and infectious etiologies, among other differentials. During the second hospitalization, the patient complained of genital ulcers, which were verified to be recurrent along with oral ulcers. The diagnosis of BD was established based on the International Classification Criteria for BD, and the symptoms improved significantly upon using colchicine and immune-suppressive drugs.

## Introduction

Behcet's disease (BD) is a rare multisystem chronic vasculitis of variable clinical presentation and unknown origin. It was first classified in 1937 and bore the name of the Turkish dermatologist, Hulusi Behcet [1]. Initially, BD was described as a triad of recurrent uveitis and oral and genital ulcers, which constitutes the major manifestations. Subsequently, several other major and minor manifestations involving the skin, joints, central nervous system, gastrointestinal tract, and large vessels were reported, indicating the multisystemic features of the disease [2-4].

The diagnosis of BD is currently based on the newly proposed International Classification Criteria for BD (ICBD), with 85% sensitivity and 95% specificity. According to these criteria, recurrent oral ulcerations, at least three episodes in a year, must be present in addition to any two of the following: (i) recurrent genital ulcerations, (ii) eye lesions, (iii) skin lesions found in adult patients not being treated with corticosteroids, and (iv) positive pathergy test read by a physician within 24-48 hours of testing [5,6].

Pulmonary involvement in BD is uncommon, occurring in only 1% to 7.7% of the patients having various forms [7,8]. While pulmonary artery aneurysms, with or without thrombosis, constitute the most frequent pulmonary involvement, pleural effusion was described only in few cases, leading to challenging differential diagnosis, notably when it is the presenting sign. Pulmonary BD is considered as a severe manifestation with high mortality, notably due to massive hemoptysis [4,7,9-11].

Herein, we report a challenging case of a young man who presented with recurrent pleural effusion and hepatic vein thrombosis, along with recurrent oral and genital ulcers. This case is being reported with an aim to emphasize the multidisciplinary work to diagnose and treat BD and prevent its complications, as well as to highlight the role of immunosuppressive treatment in improving various associated symptoms.

## Case presentation

This is the first presentation in internal medicine and recent history.

On February 25, 2020, a 21-year-old male, smoker, was directly admitted to the Internal Medicine ward for diagnostic and therapeutic thoracocentesis after pulmonology outpatient clinic advice. He had a moderate pleural effusion that was discovered on chest X-ray, which was carried out two days ago at the gastroenterology outpatient clinic as part of the investigation for lower limb edema and dyspnea.

Recent history showed papilledema that was diagnosed six months ago in a secondary care hospital and was associated with intracranial hypertension with a normal lumbar puncture, which was treated with acetazolamide that was discontinued by the patient four months later. On January 19, 2020, i.e., two months after acetazolamide discontinuation, the patient presented voluntarily at the Ophthalmology clinic of our hospital for blurred vision. On the same day, he was referred to the Neurology clinic, where computed tomography (CT) venogram and brain magnetic resonance imaging (MRI) were performed, showing empty sella syndrome with papilledema, with no evidence of cerebral venous thrombosis. Idiopathic intracranial hypertension was suspected, acetazolamide was resumed, and follow-up visits were scheduled. Three weeks later, on February 6 and 8, 2020, the patient presented to the Gastroenterology clinic for dyspepsia, where positive occult blood in the stool was found, with high inflammatory markers. An esophagogastroduodenoscopy was carried out three days later at the Daycare unit, showing erosive gastropathy with no *Helicobacter pylori*, metaplasia, or malignancy. The patient was started on esomeprazole 40 mg twice daily for one month, and a follow-up visit was scheduled on February 23, 2020. On the day of the visit, the patient complained of lower limb edema with dyspnea of recent onset. An ultrasound Doppler of the lower limbs and chest X-ray imaging were carried out, showing moderate pleural effusion without evidence for lower limb vein thrombosis. A cardiologist consultation showed normal electrocardiogram (ECG) and echocardiogram, and a pulmonologist consultation found a negative history of tuberculosis (TB) and advised hospitalization at the Internal Medicine ward for diagnostic and therapeutic thoracocentesis.

Case history

On admission, the patient reported a three-week history of chest pain of gradual onset as the chief complaint. The chest pain was located in the right hemithorax, worsened with speech and deep inspiration, and was associated with exertional dyspnea, headache, and mild abdominal pain. The chest pain was resistant to common analgesics and had no resting position. He was single, without children, and denied any personal or family history of TB or autoimmune diseases.

Physical examination

On physical examination, the patient was conscious, alert, and oriented to time, place, and person. His vital signs were normal, including blood pressure of 155/80 mmHg, pulse 67 of pulse/min, respiratory rate of 19 breaths/min, oxygen saturation of 98, and temperature of 37.1°C. The pain score was evaluated as the VAS (visual analog scale) score = 7/10. Chest examination showed slightly diminished breathing sound in the right lower and middle lung field, without wheezing or rales. Cardiac sounds were normal, with no murmurs, rubs, or gallops. The abdomen was soft without signs of tenderness, ascites, or hepatomegaly. Head, eyes, ears, nose, and throat examination showed multiple oral aphthous ulcers on the tongue and buccal mucosa. Furthermore, no enlarged lymph nodes were found in the cervical, inguinal, or axillary regions.

Diagnostic approach

In confirmation with the chest X-ray finding, a high-resolution CT of the chest without contrast was carried out showing moderate right-sided pleural effusion in addition to bilateral parenchymal process, more at the right side, of apparently infective etiology. Differential diagnoses were then TB, aspergillosis/fungal infection, connective tissue diseases, and mesothelioma. An ultrasound-guided thoracocentesis was carried on February 26, and a pigtail drain was placed. Pleural fluid cytology and biochemical analysis showed white blood cells of 285 cells/µL with lymphocyte predominance (51%), absence of malignant cells, pH = 8, adenosine deaminase (ADA) level = 7.6 IU/L (normal below 9 IU/L), and lactate dehydrogenase (LDH) = 97 IU/L. Application of Light’s criteria, pleural fluid LDH/serum LDH = 97/183 = 0.5, and total pleural fluid protein/total serum protein = 38.6/61 = 0.6 confirmed the exudative cause of the pleural effusion. A neurologist consultation recommended another lumbar puncture, but the patient refused.

**Figure 1:**
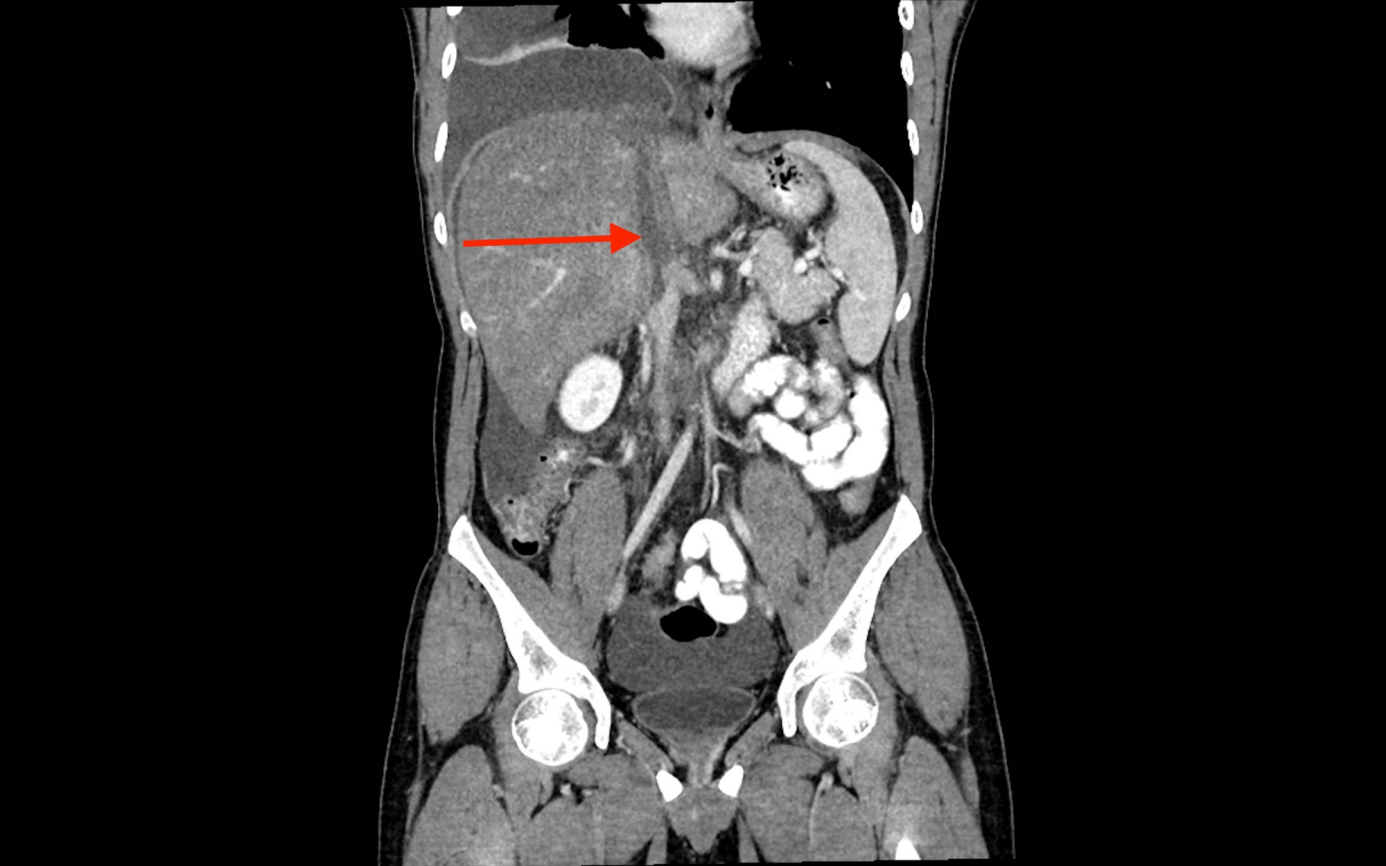
CT scan of the chest , abdomen, and pelvis with intravenous contrast , showing an IVC thrombosis. IVC, inferior vena cava

On the second day of hospitalization, the patient complained of abdominal pain. An ultrasound imaging revealed 5.5-cm inferior vena cava (IVC) partial thrombus occupying more than 80% of the IVC lumen, mild hepatomegaly, mild gallbladder wall thickening, and moderate ascites. Imaging was completed by abdomen and pelvis CT scan, as shown in Figure 1, which evidenced an extended, occlusive thrombus of the intrahepatic portion of the IVC with Budd-Chiari syndrome picture. Imaging modalities and their findings are depicted in Table 1.

**Table 1:** Imaging modalities and their findings‎ CT, computed tomography; GI, gastrointestinal; HRCT, high-resolution computed tomography; IV, intravenous; IVC, inferior vena cava; MRI, magnetic resonance imaging

Modality	Findings
Upper GI endoscopy with biopsy	Normal esophagus, diffuse erosive gastropathy, and normal duodenum; biopsy of gastric mucosa showed features suggestive of mild reactive gastropathy; no Helicobacter pylori identified; no evidence of intestinal metaplasia, dysplasia, or malignancy seen
Chest X-ray	Filling of right costophrenic angle and evocative of pleural effusion, with no left pleural effusion
CT venogram	No evidence of venous thrombosis
Brain MRI	Empty sella syndrome with papilledema, probably in relation to intracranial hypertension; no evidence of thrombosis
HRCT of the chest without contrast	It marked right pleural effusion with consolidation, bilateral pleuroparenchymal process more at the right side of the infective aspect
Ultrasound of the abdomen	Partial thrombus within the intrahepatic portion of the IVC measuring about 5.5 cm, attenuating more than 80% of the IVC lumen, with mild hepatomegaly, mild gallbladder wall thickening, and mild ascites
CT of the chest, abdomen, and pelvis with IV contrast	Large intrahepatic IVC and non-occlusive thrombus extending to the left hepatic vein with enlarged, engorged liver: findings suggestive of Budd-Chiari syndrome

Blood tests showed moderate anemia (hemoglobin = 11.1 g/dL), few reactive lymphocytes on blood smear, and moderately elevated plasma ammonia (57 µg/dL). Otherwise, blood ionogram and renal and liver function tests were unremarkable. Investigations for autoimmunity, human immunodeficiency virus (HIV) serology, and systemic inflammatory status were unremarkable except for a moderate elevation of C-reactive protein (CRP). Thrombophilia screening was negative. Other lab results included negative TB polymerase chain reaction (PCR) and interferon-gamma, in addition to normal white blood cell flow cytometry and fecal calprotectin concentration (5.9 μg/mg), ruling out lymphoproliferative disorders and inflammatory bowel disease respectively. Lab findings are depicted in Table 2.

**Table 2:** Blood tests and their findings ALP, alkaline phosphatase; ALT, alanine aminotransferase; ANA, antinuclear autoantibodies; ANCA, antineutrophil cytoplasmic antibodies; AST, aspartate aminotransferase; β2, beta-2; C-ANCA, cytoplasmic anti-neutrophil cytoplasmic antibody; CCP, cyclic citrullinated peptide; CRP, C-reactive protein; DNA, deoxyribonucleic acid; DS-DNA AB, double stranded-deoxyribonucleic acid antibody; ELISA, enzyme-linked immunosorbent assay; ESR, erythrocyte sedimentation rate; GGT, gamma-glutamyl transferase; HIV; human immunodeficiency virus; HLA, human leukocyte antigens; Ig, immunoglobulin; LDH, lactate dehydrogenase; HLA B27, human leukocyte antigen B27; P-ANCA, perinuclear anti-neutrophil cytoplasmic antibody

Investigation	Findings	Normal Range
Hematology	Hemoglobin: 11.1 g/dL	13.5-17.5 g/dL
	Platelets: 360 K/mm^3 ^	140-450 K/mm^3^
	Blood smear: few reactive lymphocytes	
Biochemistry	Sodium:140 mEq/L	135-147 mEq/L
	Potassium: 4.6 mEq/L	3.5-5 mEq/L
	Ammonemia: 57 µg/dL	0-93 µg/dL
Renal function	Creatinine: 0.8 mg/dL	0.62-1.42 mg/dL,
	Glomerular filtration rate:118 mL/min/1.73 m^2^	
Liver function	AST: 13 IU/L	0-45 IU/L
	ALT: 14 IU/L	10-45 IU/L
	GGT: 89 IU/L	8-61 IU/L
	ALP: 133 IU/L	40-115 IU/L
	LDH: 174 IU/L	35-225 IU/L
	Bilirubin total: 1.15 mg/dL	0-1.52 mg/dl
Inflammatory markers	ESR: 4 mm/hour	0-15 mm/hour
	CRP: 6 mg/dL (moderately elevated)	0-5 mg/dL
Immunology and serological testing	HIV antibodies: negative	Negative
	ANA ELISA: 7.01 IU/mL; ANA quantitative immune fluorescent assay titer: <1:40	Negative
	Anti-double-stranded DNA: negative	Negative
	Anticardiolipin IgM and IgG: negative	Negative
	Anti-β2-glycoprotein I IgG and IgM: negative	Negative
	C-ANCA and P-ANCA: Negative	Negative
	HLA B27: negative	Negative
	Interferon-gamma release essay: negative	Negative
	Cryoglobulin: negative	Negative
	Anti-CCP: 7 IU/mL	0-19 IU/mL
	Rheumatoid factor: 4.2 IU/mL	0-14 IU/ml
Thrombophilia	Factor V Leiden mutation: absent	Absent
	Prothrombin gene mutation: absent	Absent
	Antithrombin III: normal	Normal
	DS-DNA AB 14: negative	Negative
	Anti β2-glycoprotein antibodies: negative	Negative
	Lupus anticoagulant: normal	Normal

The patient was started on enoxaparin 50 mg twice daily subcutaneous, and updated differentials were vasculitis, lymphoma, and pleural TB. A multidisciplinary meeting was held, during which myeloproliferative disorder was added to the differentials, and a bone marrow biopsy was indicated. Regarding the pleural effusion, video-assisted thoracotomy (VAT) biopsy was discussed, notably in the case of recurrent fluid accumulation and in the absence of an etiological diagnosis.

After one week of hospitalization, the fluid collection was minimal, and the patient was otherwise asymptomatic. The pleural pigtail was removed, and the patient was discharged on enoxaparin 60 mg twice daily subcutaneous for 20 days. Follow-up visits at the Thoracic Surgery, Hematology, Pulmonology, Gastroenterology, and Infectious Diseases clinics were scheduled.

Second admission

After the first discharge, the patient rapidly accumulated right-sided pleural effusion that was evidenced on chest X-ray imaging during the pulmonology follow-up visit. The patient was readmitted electively on March 8, 2020, i.e., four days after discharge from the first hospitalization. During the second hospitalization, VAT biopsy was carried out on March 11. Histological examination showed mainly chronic inflammatory changes with no evidence of granuloma. Microbiologically, cultures were negative, and three mycobacterium TB direct tests and cultures were negative in pleural biopsy samples, and no fungal growth was noted.

Inpatient gastroenterologist's follow-up of ascites and Budd-Chiari syndrome recommended a fibro scan, which showed 49.6K pascal fibrosis, indicating stage 4 liver cirrhosis. Treatment with spironolactone 25 mg once daily orally and furosemide 40 mg once daily orally was started.

During this hospitalization, the patient requested topical treatment for genital sores, which were found to be genital ulcers. The patient was seen by a rheumatologist, who conducted a deeper history investigation showing a five-year history of recurrent, painful oral ulcers, weight loss of approximately 6% in six months, and genital ulcerations occurring after shaving. Based on these observations, the diagnosis of BD was made according to the ICBD criteria. Thus, the pleural effusion was attributed to BD, with regard to the negative workups of TB and malignancy. The patient was started on colchicine 0.5 mg twice daily orally and prednisolone 30 mg once daily orally.

On March 22, the patient was discharged on the same doses of colchicine and prednisolone for 30 days, the same dose of enoxaparin for 10 days, acetazolamide 500 mg twice daily for 30 days, warfarin 4 mg once daily for 10 days (until reaching a therapeutic international normalized ratio [INR] between 2 and 3.5), furosemide 20 mg once daily for 30 days, and spironolactone 25 mg orally once daily. Additionally, a multidisciplinary follow-up, including rheumatology, ophthalmology, pulmonology, neurology, gastroenterology, hematology, and thoracic surgery, was scheduled.

Follow-up

On March 31, 2020, significant improvement of oral ulcerations was noted by the rheumatologist, and azathioprine 50 mg once daily was added. No re-accumulation of pleural effusion was observed on further chest-X rays. Warfarin dose was increased to 4 mg orally three times daily due to unreached therapeutic INR.

On April 14, 2020, prednisolone was decreased with a tapering plan over eight weeks.

After three months of follow-up, no evidence of pleural effusion was found on chest X-ray imaging, and the patient did not complain of pain or oral or genital ulcer recurrence.

## Discussion

The diagnosis of BD in our case was challenging due to the uncommon presentation, including exudative pleural effusion associated with IVC thrombosis and Budd-Chiari syndrome. In addition, the presence of intracranial hypertension with papilledema in recent history made the clinical picture even more complex and confusing. The initial non-conclusive investigations and multidisciplinary discussions were guided by the suspicion of a malignant or infective etiology.

Pleural effusion is common in IVC or hepatic diseases, notably cirrhosis, and it is explained by the development of portal hypertension [12]. Therefore, we might hypothesize that the pleural effusion in our patient was due to the portal system transudate as he had both cirrhosis and IVC thrombosis. However, in such conditions, the pleural fluid is transudative; whereas, in our patient, the fluid was exudative by application of Light’s criteria. Thus, pleural effusion is believed to be linked to BD, as one of the rare pulmonary manifestations [4,7,9-11].

Initially, the exudative feature of the pleural effusion suggested a local factor, which indicated the pleural biopsy to rule out mesothelioma or an infection such as TB or aspergillosis [13]. Further differentials were mentioned, such as autoimmune diseases, thrombophilia, and lymphoproliferative disease; all have been ruled out by specific investigations, and the patient was treated symptomatically.

As a presenting symptom, pleural effusion is extremely rare in BD. The only case found in history is of a 24-year-old male who presented with a massive chylothorax occupying all the right hemithorax. The patient consulted at the emergency department for a three-month history of dyspnea, low-grade fever, nonproductive cough, and noted recurrent, painful genital and oral ulcers. Further investigation showed multiple thromboses, including the right internal jugular and right subclavian veins, the innominate vein, and the superior vena cava [14]. The association of these central venous thromboses with the recurrent oral and genital ulcers reported by the patient made the diagnosis of BD more easily suspected. By comparison, although these clinical manifestations were found in our case, the diagnosis of BD was more challenging due to the patient not reporting recurrent oral or genital ulcers in his history. Furthermore, genital ulcers were incidentally discovered as the patient requested topical treatment without specifying the localization of his lesion until he was interrogated. This is probably due to cultural factors, where patients’ modesty may hinder the clinical approach.

On the other hand, oral ulcers evidenced during the physical examination might have theoretically led to the suspicion of systemic disease. However, due to focus on malignancy and infectious etiologies and the patient not initially reporting a recurrence of these oral ulcers, these were considered as aphthous stomatitis and attributed to common factors such as smoking and stress, without suspecting a systemic vasculitis [15,16]. This highlights the importance of considering the investigation of minor clinical symptoms, such as oral ulcers, especially in case of challenging clinical picture.

Subsequent to treatment, the patient reported satisfactory regression of all symptoms, and investigation showed no recurrence of pleural effusion after three months of follow-up. BD is a chronic disease that presents as recurrent attacks of unknown etiology, which is spared with remission phases of variable duration. Consequently, the main objective of treatment is to maintain remission and improve patient’s quality of life. In general, anti-inflammatory and immunosuppressive drugs are adopted due to the inflammatory nature of the disease [2,5,17,18]. However, depending on the organ involved and case severity, the treatment choice can vary. Although no guidelines are established for the management of BD, a corticosteroid with or without anticoagulant have been used in the treatment of pulmonary involvement. Additionally, clinical trials have proved the efficacy of colchicine, a well-known anti-gout drug for the treatment of BD [19,20].

## Conclusions

This is a rare case of exudative pleural effusion with central venous thrombosis presenting in a patient with BD, initially suggesting malignant or infectious etiology and requiring multiple investigations. The diagnosis was made after the incidental detection of genital ulcers that were not initially reported by the patient and which revealed to be recurrent. The treatment using colchicine and immune-suppressive drugs was efficient in improving the symptoms.
